# Assessing the Rheological Properties of Durum Wheat Semolina: A Review

**DOI:** 10.3390/foods10122947

**Published:** 2021-11-30

**Authors:** Cristina Cecchini, Andrea Bresciani, Paolo Menesatti, Maria Ambrogina Pagani, Alessandra Marti

**Affiliations:** 1 Consiglio per la Ricerca in Agricoltura e L’analisi Dell’economia Agraria (CREA), Centro di Ricerca Ingegneria e Trasformazioni Agroalimentari, Via Manziana 30, 00189 Rome, Italy; cristina.cecchini@crea.gov.it; 2 Department of Food, Environmental and Nutritional Sciences (DeFENS), Università Degli Studi di Milano, Via G. Celoria 2, 20133 Milan, Italy; andrea.bresciani@unimi.it (A.B.); ambrogina.pagani@unimi.it (M.A.P.); 3 Consiglio per la Ricerca in Agricoltura e L’analisi Dell’economia Agraria (CREA), Centro di Ricerca Ingegneria e Trasformazioni Agroalimentari, Via Della Pascolare 16, Monterotondo, 00015 Rome, Italy; paolo.menesatti@crea.gov.it

**Keywords:** durum wheat, semolina, gluten quality, protein network, rheology, pasta

## Abstract

Empiric rheology is considered a useful tool for assessing the technological quality of wheat. Over the decades, several tests have been adapted from common to durum wheat, and new approaches have been proposed to meet the needs of the players of the durum wheat value chain. Breeders are looking for reliable methods to test the functional quality of wheat lines at early stages, where there are limited amounts of sample; millers need fast and reliable methods for checking wheat quality right at the point of the receiving station; and pasta-makers are looking for suitable methods to predict end product quality. This review provides an overview of the strengths and weaknesses of the rheological tests currently used to evaluate the quality of durum wheat semolina, with the emphasis on Europe. Moreover, the relationships among the parameters obtained from different rheological approaches are extrapolated from the literature and integrated with the data obtained from 74 samples of durum wheat semolina. Although numerous efforts have been made to propose rapid and reliable tests for semolina characterization, the ideal test has yet to be proposed, indicating that researchers and pasta companies need to focus on perfecting the way to assess the quality of durum wheat and pasta.

## 1. Introduction

Durum wheat semolina is considered the ideal raw material to produce dry pasta; this statement is well accepted by all the players of the durum wheat value chain, from breeders to pasta-makers and consumers. This is true not only in Italy, Greece, and France—where only semolina can be used to produce dry pasta legally—but also outside of the Mediterranean area. Specifically, the suitability of semolina for pasta-making is due to the ability of the corresponding dough to withstand the numerous physical stresses occurring during processing [1]. This property is mainly due to the quantity and quality of its protein fractions. Indeed, the combination of protein quantity and quality results in—after cooking—a continuous and coagulated protein network that surrounds the gelatinized starch granules. As described by Resmini and Pagani [2] in the 1980s, on the basis of ultrastructure observations and confirmed in more recent years by several authors [3,4,5], pasta is considered to be of good quality, i.e., with high firmness (i.e., the degree of resistance to the first bite) and no stickiness (i.e., the adhesion rate of pasta to tongue, teeth, palate, and/or fingers) and no or minimal bulkiness (i.e., the adhesion rate of cooked pasta strands among them), if after cooking, a three-dimensional, continuous, almost non-deformable and elastic protein network surrounds each starch granule. This optimal structure is guaranteed if proteins coagulate before starch swelling (due to the large availability of water and high temperatures), so that the starchy material will remain mostly trapped within the protein network, with negligible amylose release into the cooking water and equally limited quantities of amylopectin on the surface of pasta [3,6].

The quality of raw materials and processing conditions are responsible for the cooking behavior of pasta. The role of drying temperature in enhancing the formation of a regular protein structure around the starch granules has been elsewhere described [5,7,8,9,10,11]. However, the selection of high-quality semolina remains the objective of pasta-making companies. Although several factors contribute to the definition of “high-quality semolina”, the focus of this review is the evaluation of the technological quality, i.e., the tendency to form the peculiar structure described above that leads to the typical “al dente” firmness of cooked pasta. Various approaches have been proposed—from molecular to macroscopic—to assess the technological quality of durum wheat semolina. In this context, the present work summarizes the main factors determining the technological quality of durum wheat semolina and reviews the main approaches widely used (especially in Italy and France) for gluten quality evaluation, indicating the strengths and weaknesses of each test. Moreover, the relationships among the parameters of the different rheological tests are presented and discussed.

## 2. Defining Gluten Quality

The amount of protein in durum wheat is the first parameter that dry pasta producers consider when choosing the raw material. According to the voluntary classification used in Italy [12] in the field of durum wheat, semolina is classified into three classes: for the lowest quality class protein content ranges from 10.5% to 11.9%; the medium class includes samples with 12.0–13.5% protein content, while the excellent semolina quality exhibits at least 13.5% protein. A high amount of protein, in fact, is the prerequisite for a dough in which the gluten matrix is sufficiently thick and well developed even in conditions of non-optimal hydration, like those used in pasta-making (i.e., 30–32% moisture content).

Although the high protein—and consequently gluten (about 30% wet basis; >11% dry basis)—content is an important quality requirement [7,10], this characteristic is not enough to guarantee the good cooking behavior of the corresponding pasta. Indeed, regardless the particle size and protein content, pasta made with common wheat differs in structure and/or firmness from that made with good durum wheat semolina. Moreover, Fuad and Prabhasankar [13] stated that the use of common wheat flour in pasta-making is associated with good cooking quality when additives and optimized technologies are used. The superiority of durum wheat over common wheat is not, in fact, only related to protein content (on average two percentage points higher than common wheat), but to the composition of protein fractions. In this regard it has been shown that the suitability of durum wheat in pasta-making is related to specific combinations of alleles at the storage protein loci: glutenin alleles at low molecular weight (LMW) locus Glu-B3 and at high molecular weight (HMW) locus Glu-B1 [14]. With regard to common wheat, HMW glutenins (HMW-GS) are crucial in guaranteeing the formation of a gluten network suitable for bread-making above all for the presence of Glu-D1 locus that is absent in durum wheat [14,15]. On the other hand, in durum wheat, the formation of a structure suitable for pasta-making is related to the high density of cross links between the shorter chains of LMW glutenins (LMW-GS) [16].

At this point it is necessary to clarify what “suitable for pasta-making” means. Several researchers have used different terms to describe the features of durum wheat gluten that mainly affect pasta quality: strength, tenacity, and elasticity (Table 1). All of them refer to the dough and/or gluten rheological properties, which describe the interactions between the different macromolecules that lead to the formation of the gluten network and, therefore, of the dough. The protein network developed during the mixing and kneading phase of pasta-processing is stabilized by both covalent bonds (disulfide bonds) and bonds of lower energy, such as hydrogen bonds and hydrophobic interactions between non-polar amino acid residues [17,18,19]. The adjective “strong” is often referred to gluten characterized by high tenacity and/or strength, whereas the adjective “weak” is used to describe gluten with low tenacity and/or strength, and high extensibility.

## 3. Assessing Gluten Quality

Dough is one of the most difficult materials to characterize from a rheological point of view [20]. In fact, it exhibits viscoelastic behavior defined as plastic by Bushuk [21], or in other words, its behavior ranges between that of an elastic solid and that of a viscous liquid. Moreover, the characteristics of dough change at each stage of the process (especially due to temperature changes occurring during pasta-making) and it is therefore difficult to predict its behavior during processing. This complexity justifies the development of so-called “empirical” rheological tests, which are widely used in the industry. In any case, as pointed out by Dobraszczyk [20], it is essential to define, for each processing variable (humidity, temperature, pressure, etc.), the range of values applied in the step/phase that is under investigation.

**Figure 1:**
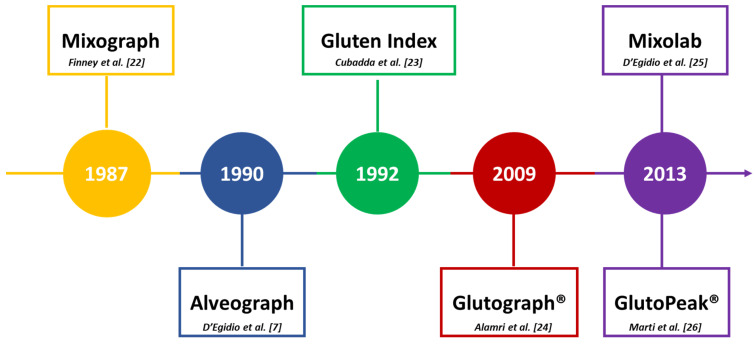
Time sequence of the rheological tests adopted in the durum wheat value chain [7,22,23,24,25,26].

Since the 1980s, several rheological tests have been proposed to characterize durum wheat semolina and to objectively describe its pasta-making performance, as summarized in Figure 1.

In general terms, the tests used for the rheological characterization of durum wheat can be classified according to various criteria. Some of them (i.e., Gluten Index and Glutograph® tests) directly evaluate the quality of gluten after its extraction from semolina, while others are carried out on dough (i.e., Alveograph, Mixograph, and Mixolab tests) or slurry (i.e., GlutoPeak test) systems. Some of them provide information mainly about strength (i.e., Mixograph, and Mixolab tests), others also provide details on extensibility (e.g., Alveograph) or elasticity (e.g., Glutograph® test). Some of them (i.e., Alveograph, GlutoPeak) test sample breakage, others do not (i.e., Glutograph®, Mixograph, Mixolab). Some of them are used more in Europe than in the United States or Canada, and vice versa, depending on the country of the company that produced the device. For example, the Alveograph is mostly used in European countries, whereas the Mixograph is widely used in North America [27]. As the present review is focusing on the rheological approaches used in Mediterranean countries, the use of the Mixograph for semolina characterization will not be addressed. For further information, readers should see the research of Dick and Youngs [28], Rath et al. [29], Finney [30], Khatkar et al. [31], Kovacs et al. [32], and AbuHammad et al. [33].

The main approaches used for semolina characterization are summarized in Table 2. From the well-known methods to the most recent, the common goal has been to respond to the needs of the operators of the supply chain who are often asked to provide, as quickly as possible, a reliable prediction of the behavior of the raw material during both the pasta-making process and cooking. In particular, breeders need to analyze in a short time a very large number of new breeding lines and released varieties, for which the quantity of material represents a limiting factor [34]. The milling industry needs fast, simple, and reliable methods to control the quality of wheat during the reception phase, in terms of milling yield and semolina characteristics that define its commercial value. Finally, the pasta industry also needs rapid and reliable methods that determine the pasta-making ability of the semolina and predict the cooking quality of the finished product. In this context, near infrared (NIR) spectroscopy, a rapid and non-destructive technique widely used in the industry to determine moisture and protein content [35], is becoming increasingly studied as a technique to predict some of the indices expressed by rheological tests to define the technological quality of semolina [36,37]. Nevertheless, the NIR prediction of qualitative rheological parameters requires robust calibration models to extract information from the spectral data [38].

Most of the tests were proposed for the common wheat sector, so they do not simulate, either by type or intensity, the stresses that arise during pasta extrusion and drying. In some cases, the method has been adapted to measure the quality of durum wheat by making some modest/small changes (e.g., resting time of the dough in the Alveograph). It follows that the information gathered from the current tests is mainly useful for classifying semolina in broad classes (excellent, good, or poor gluten quality), whereas the screening of samples within each class is still challenging. Although the limitations of such tests are well known, most of them are widely used, as a reference in the industry, to predict the pasta-making quality of durum wheat semolina [7,10,34,39,40,41,42].

Basically, the procedures have not changed over the years, but additional data integration systems have been developed for directly processing the values of the parameters provided by the instruments. A brief description of each test will be provided in the following sections, whereas the main indices provided by each test are reported in Table 3.

**Table 2:** Rheological approaches used for semolina characterization.

Test	Principle	Hydration Level	Features	Standard Method for Durum Wheat Semolina
Gluten Index	Gluten ability to pass through a sieve after centrifugation	not required	- Short time for analysis (10 min) - Small amount of sample (10 g) - Need to extract gluten - Overestimation of the value in case of low protein content samples - Low capacity of discriminating semolina of medium quality	Yes [43,44]
Glutograph®	Gluten resistance to stretching	not required	- Short time for analysis (20 min, including extraction and resting time) - Small amount of sample (10 g) - Need to extract gluten - High variability	No
Alveograph	Dough resistance to tridimensional extension	≈52 g water/100 g semolina (14% moisture basis)	- Long time for analysis (50 min) - Large amount of sample (250 g) - High influence of the analyst - Widely used in the field, especially in Europe	Yes [45]
GlutoPeak®	Aggregation kinetics of gluten proteins	≈100 g water/100 g semolina (14% moisture basis)	- Short time for analysis (5-10 min) - Small amount of sample (9 or 10 g) - Low influence of the analyst - Few available studies	No
Mixolab	Dough resistance to both mechanical and thermal stress	≈60 g water/100 g semolina (14% moisture basis)	- Long time for analysis (45 min) - Large amount of sample (50 g) - Low influence of the analyst - Difficulty in following the set temperature profile	No

### 3.1. Approaches Using Extracted Gluten

Gluten Index and the Glutograph® test have in common the short time required for analysis (about 10–20 min), the small quantity of sample (10 g) and their applicability even to whole grain flours, eliminating the refinement process to obtain semolina. While providing useful information on specific properties of gluten, both tests might underestimate the effect of other wheat constituents and their interactions with proteins because they are measuring extracted gluten. On the other hand, such interactions might affect sample behavior during the pasta-making processing, and thus the quality of the final product. Although some researchers have highlighted high variability related to the extraction phase, both tests have great potential for use in breeding programs.

#### 3.1.1. Gluten Index

The Gluten Index (GI) is a measure of the quality of gluten after mechanical extraction at room temperature: the higher the value, the stronger the gluten. This method is widely used for the screening of durum wheat varieties based on gluten strength [23], as well as in international trade specifications [27]. Briefly, wet gluten is extracted (using the Glutomatic® instrument, Perten part of Perkin Elmer, Waltham, MA, USA) from semolina by washing both starch and soluble proteins with a sodium chloride solution during mechanical mixing to form the gluten. After which the wet gluten is centrifuged to force it through a specific sieve under standardized conditions: the percent of gluten that remains above the sieve corresponds to the GI. Durum wheat and/or semolina with GI values higher than 80 are the preferred raw materials to produce high quality pasta [33]. A negative correlation was found between GI and both gliadin content and the gliadin to glutenin ratio [46,47,48]: semolina with high gliadin/glutenin ratios are more extensible, resulting in low GI values. On the other hand, a strong relationship of GI to unextractable polymeric protein was found [47,48,49].

Conflicting results were found for the relationship between GI values and HMW-GS/LMW-GS ratio: Sissons et al. [47] highlighted a positive correlation, while Edwards et al. [49] found that high proportions of HMW-GS consistently corresponded with low GI values. These findings are in agreement with the statement that in durum wheat the formation of a well-developed network would preferentially involve LMW-GS over HMW-GS: the shorter chain lengths result in greater density of cross links for a given volume and therefore impart greater strength [16].

GI is relatively independent of protein content [10,41]. On the other hand, a negative correlation was found between dry gluten content and GI (average value of r = −0.506; p < 0.01; [50]) with a possible over-estimation of the index itself, probably attributable to purely mechanical causes. A lower gluten mass encounters a lower centrifugal force as compared to a higher gluten mass (thus a higher percentage of wet gluten remained on the sieve), resulting in a higher GI [51]. Furthermore, some authors report that samples characterized by a very tenacious gluten (such as durum with Glu-D1 HMW-GS 5+10) often fails to form a gluten ball, so this test is able to give no data [52].

Another drawback of this method is its low capacity to differentiate semolina of medium quality. Indeed, for semolina samples with a GI in the 30–65 range, the GI did not show any significant correlation with quality attributes (i.e., firmness, stickiness, bulkiness, and overall quality) of pasta dried using a low temperature drying cycle [42].

Interestingly, the test is not influenced by the extraction rate of the semolina: indeed, although the higher the extraction rate, the higher the protein content, proteins present in bran are not gluten proteins [53]. More recently, the test was successfully used to assess the quality of old cultivars compared to modern ones: the latter showed stronger gluten than the former due to both their genotypic and phenotypic differences [54].

#### 3.1.2. Glutograph®

**Figure 2:**
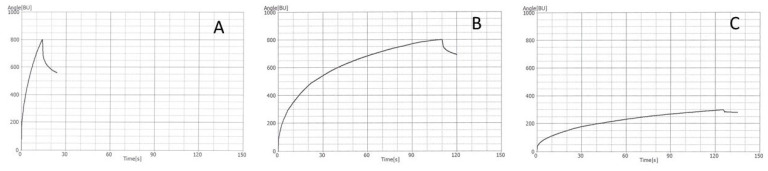
Glutograph® profile of semolina with poor (**A**) and good (**B**,**C**) quality. Strong gluten (**B**): stretching angle expressed in seconds; very strong gluten (**C**): stretching angle expressed in Brabender units (BU).

The Glutograph® (Brabender, Duisburg, Germany) device measures the extensibility and elasticity of gluten quantifying its resistance to stretching and its recovery. Although widely used by industries to evaluate semolina quality, the Glutograph® is rarely mentioned in the literature. The measuring system of the instrument consists of two parallel, round, finely corrugated plates set at a pre-determined distance. The analytical conditions for this test are those indicated in the manufacturer’s manual (Brabender, Germany) as there are no official methods. During the test, while the upper plate remains still, the lower plate is turned with a constant moment till a fixed angle is reached (i.e., 800 BU) (stretching phase). This constant force determines the deformation of the dough. After stretching, the force is released for 10 s (relaxing phase) and the sample contracts according to its elasticity. Strong gluten requires prolonged “stretching” times and low relaxation values compared to weak gluten (Figure 2). Based on the results of the present study and in agreement with those obtained by AbuHammad et al. [33], very strong gluten exhibits stretching time > 75 s, whereas values between 30 and 74 s are typical of strong gluten. On the other hand, moderately good gluten and weak gluten show stretching time of 12–29 s and <11 s, respectively.

On the other hand, relaxation values are not a good indicator of gluten strength and cannot be used to differentiate cultivars according to their gluten quality.

Moreover, in case of tenacious gluten, poor repeatability and reproducibility of the results are observed. Furthermore, for very tenacious gluten the stretching angle (800 BU) is not reached during the shear phase (Figure 2C). In this case the result is counted as the stretching angle reached at the stretch abort time (125 s) and is expressed in BU. Therefore, the results of strong gluten expressed with different units (for example, the samples in Figure 2B,C) are difficult to compare. In addition, an unusually high coefficient of variability for the indices was observed compared with other parameters, likely due to either the high level of variability among cultivars or execution of test procedures [33].

### 3.2. Approaches on Dough System

Although using different hydration levels, the Alveograph and the Mixolab provide information on dough behavior during specific stresses (Table 2). The Alveograph, while requiring a large amount of semolina (250 g), is widely used internationally in the rheological characterization of doughs due to its ability to simultaneously define dough strength and extensibility. Both approaches require a long run time for each sample (45–50 min) which makes them unsuitable for rapid evaluation of gluten quality, as required by the industry and breeding programs.

#### 3.2.1. Alveograph

The Alveograph test (Chopin, Villeneuve-la-garenne, France) was developed for the characterization of common wheat flour. Widely used in Europe, it evaluates dough resistance to three-dimensional expansion, thus simulating biological leavening and, therefore, the development dough volume due to the accumulation of carbon dioxide produced by yeasts. Nevertheless, it can be applied to durum wheat semolina by increasing the kneading time (from 8 to 26 min). In this case, the Alveograph test could provide information on the ability of the dough to withstand mechanical stress during pasta processing. The pressure promoted by air insufflation that is necessary for the blowing—until breakage—of a dough disc, is measured and recorded as an alveogram, yielding the indices reported in Table 2.

**Figure 3:**
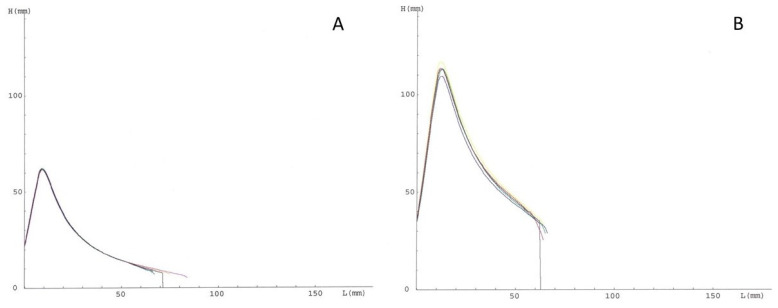
Alveograph profile of semolina with poor (**A**) and good (**B**) quality. L, length; H, height.

Figure 3 reports an example of graphs for semolina samples with poor (Figure 3A) and good (Figure 3B) pasta-making performances. In the pasta-making sector, high P/L values (i.e., >1) are associated with strong gluten, while low values (i.e., <0.5) indicate weak gluten, not suitable for pasta production.

The W value can be considered a measure of the gluten network quality: high W values are associated with the formation of a strong network able to retain starch granules during cooking. Therefore, the W value is considered a valid parameter to predict the cooking quality of pasta [7,32,33]. Recently, Phakela et al. [55] found a negative correlation between both the HMW glutenins and α- and ω-gliadins with dough strength (W). On the other hand, W was positively correlated with the γ-gliadins. As regards dough extensibility (L), it was negatively correlated with LMW-GS.

The test is carried out at a constant level of hydration. In the case of durum wheat dough, this aspect might be critical since it does not consider the influence of some characteristics of the raw material (including protein amount and damaged starch content) on dough consistency and its ability to absorb and retain water [56]. Thus, in the case of strong flours, the high P value might be due to the incompletely and insufficiently homogeneous hydrated protein matrix. The hydration level reached in the Alveograph test (about 52% for semolina with 14% moisture content) does not guarantee the complete hydration of durum wheat proteins but is closer to the moisture used in pasta processing (water is added to semolina to obtain a mass of 30–32% moisture).

#### 3.2.2. Mixolab

**Figure 4:**
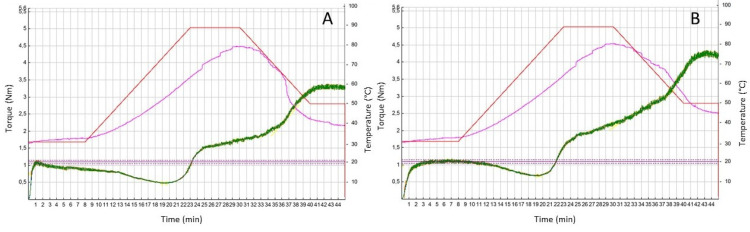
Mixolab profile of semolina with poor (**A**) and good (**B**) quality. Green line: torque; red line: temperature profile; purple line: dough temperature.

Mixolab (Chopin, France) is used to measure the rheological properties of a dough subject simultaneously to mechanical kneading and heating with a temperature gradient. This approach is potentially capable of giving information on both protein and starch properties in a single analysis [57]. In addition to the “water absorption” index—which is of particular interest for common wheat—the test provides indications on dough behaviour during mixing, therefore, on the strength of the gluten network, on the effect of amylase activity, as well as on the gelatinization and retrogradation of starch (Figure 4). The method was initially developed for the evaluation of dough from common wheat flour, but it was also adapted to characterize durum wheat semolina [25]. The correlations between other rheological tests and Mixolab were reported by D’Egidio et al. [25], particularly the parameters related to the protein component (stability, C2 and C1–C2) showed a correlation with protein content and Alveograph W. Good quality semolina samples show higher stability during mixing than poor semolina (Figure 4).

Protein and starch are related to each other as the intensity of the gelatinization of starch is inversely related to the protein and gluten content. The swelling rate and gelatinization level both depend on the availability of water in the dough [58], as well as the formation of the gluten network, whereas a higher protein level could signify less water availability for starch gelatinization [25].

Recently, Mixolab® has been successfully used to quickly detect the damage caused by sunn pests in durum wheat [59]. Moreover, Torbica et al. [60] applied the Mixolab to characterize fourteen durum wheat breeding lines grown during two production years with different climate conditions: genotypes greatly affected indices related to protein quality, while the production year influenced indices related to starch.

### 3.3. Innovative Approach: The GlutoPeak®

GlutoPeak® (Brabender, Germany) has recently been proposed for the evaluation of wheat quality by determining the aggregation properties of gluten. Compared to conventional tests (Table 2), the analysis performed with GlutoPeak® has several advantages, in terms of the quantity of sample required (<10 g), analysis time (<5 min), ease of use and operator influence (very low). It measures the aggregation behaviour of gluten when water (in excess) is added and mixed at a high speed (up to 3000 rpm).

**Figure 5:**
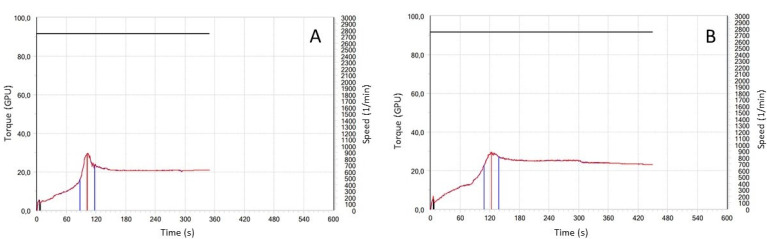
GlutoPeak profile of semolina with poor (**A**) and good (**B**) quality.

The curve is characterized by an increase in consistency up to a peak (also called BEM, and expressed in GlutoPeak Units, GPU) that corresponds to the maximum gluten aggregation. The time of maximum consistency is called peak maximum time (PMT). After this point, the consistency decreases following the breaking of the gluten network due to intense mechanical action. Generally, low values of BEM and PMT indicate poor aggregation properties, and thus low pasta-making performance [26,61], as reported in Figure 5.

Until now there has been no official methodology for this test. From the literature it emerges how each researcher adopts different analysis conditions (i.e., sample:water ratio, paddle speed, temperature, and solvent type) and that the latter can change according to the type of sample. It is therefore difficult to compare the results obtained from different papers and their interpretation. For example, Marti et al. [42], when proposing the use of the energy index for the first time (area underlying the curve, up to 5 min), expressed this index in arbitrary units. This parameter could discriminate semolina on the basis of pasta performance [42]. With updated software, the energy index is automatically calculated and expressed as aggregation energy (i.e., the area under the curve 15 s before and 15 s after the BEM) or as total energy (i.e., the area under the curve from the beginning until 15 s after the BEM). More recently, Sissons and Smit [62] proposed the use of an index not currently provided by the software but interesting to evaluate gluten strength: the gluten strength index (GSI), obtained as a product of BEM and total energy.

As regards applications, Grassi et al. [61] adopted the GlutoPeak test for differentiating durum wheat cultivars based on the glutenin to gliadin ratio. Specifically, high and medium-high quality varieties were differentiated from those of low and medium-low quality based on the aggregation energy and BEM.

Some authors [62,63] have tried to optimize the analysis conditions regarding the paddle rotation speed (1900–2700) and the semolina:water ratio (7/10:10). The best results, in terms of low coefficients of variation for the PMT and total energy indices, were obtained using semolina:water ratio of 9:10 and a rotation speed of 2700 rpm. Lower rotation speeds (for example 1900 rpm) are better to discriminate semolina samples of different strengths, but they result in higher variability [62].

When applied to wholemeal semolina, the GlutoPeak test could be considered as a useful tool in the genetic selection phase of durum wheat lines [62]. Wholemeal semolina showed a shorter PMT and a higher BEM compared to refined semolina; however, significant correlations were found for PMT (r = 0.816), total energy (r = 0.814), and GSI (r = 0.804) indices obtained from refined semolina and those obtained from wholemeal [62]. The analysis on wholemeal would further reduce analysis times related to the preparation of the refined sample in genetic selection studies. In addition, the use of wholemeal would limit the variability linked to semolina particle size, an aspect of great importance in the durum wheat sector.

### 3.4. Non-Rheological Approach: The Sedimentation Test

Although this review is an overview of rheological tests to evaluate the quality of gluten, we cannot omit the sodium dodecyl sulphate (SDS) sedimentation test, still one of the most popular and used approaches. Unlike the other tests previously described, it is a physical–chemical method that provides indications on the quantity and quality of those protein fractions that define the characteristics of gluten. The test, in fact, is based on the property of gluten proteins to swell and flocculate in an acid medium. Under specific conditions, a suspension of wholemeal in a lactic acid-SDS solution forms a sediment whose volume represents the sedimentation index [63,64]. When the volume (or index) is high, the sedimentation is slow, and the quality of the flour is better. SDS values of 30–40 mL indicate good quality gluten, and values greater than 40 mL indicate excellent quality and, therefore, strong gluten [33].

The SDS test was initially developed for the evaluation of the baking quality of common wheat [64]. Subsequently, Dexter et al. [65] applied the test on durum wheat: by increasing the SDS concentration, the absolute values changed, but the qualitative differences among the samples were maintained. The increase in the SDS concentration therefore promotes greater differences in sedimentation volumes among durum wheat of different quality, allowing better differentiation among samples of similar quality.

The test is commonly used as a rapid method in quality controls and to predict gluten quality in wheat selection programs in early generations when the quantity of seed is a limiting factor. The test, in fact, requires a lower quantity of sample (6 g) and fewer manual skills; a “micro-method” was also developed, applicable on just 1 g of wholemeal flour [66].

## 4. Relation among the Main Rheological Approaches and Relevance for Cooking Quality

Assessing the quality of gluten is an old but still relevant topic. Indeed, the selection of new lines/varieties as well as the impact of climate change on the quality of wheat crops and, consequently, on gluten quality, account for the number of studies evaluating the pasta-making potential of durum wheat samples. At the same time, several studies aimed at correlating the parameters obtained from different rheological approaches. Such studies are certainly not a pure publication exercise, but are driven by various aspects, for example:

(I) Every time a new device appears on the market, it is necessary to verify its reliability by correlating its indices with those obtained by well-established, conventional approaches;

(II) Each rheological approach provides information on a specific gluten attribute (e.g., elasticity, tenacity, extensibility, and strength); hence, the need to evaluate semolina quality by using all the available rheological tests and/or to find one approach that in the shortest possible time provides information that can be correlated to as many attributes/indices as possible.

Thus, Pearson coefficients and their significance have been extrapolated from the studies on the rheological properties of durum wheat semolina and summarized in Table S1. The bibliographic data were integrated with the results obtained by applying the main tests described in the previous section (i.e., Gluten Index, Glutograph®, Alveograph, GlutoPeak®, and SDS) to a set of 74 samples of durum wheat semolina from Italian experimental trials of varietal comparison conducted in the agricultural year 2016/2017.

SDS and GI are widely used among breeders to select durum wheat varieties [67]. If a positive correlation is found between SDS and protein content, the GI appears to be relatively independent of proteins. Both SDS and GI are significantly correlated to various parameters for evaluating the rheological quality of semolina, but the most interesting correlations were observed with the Alveograph indices [7,10,32,34]. Nevertheless, as genotype x environment interaction significantly affects both Alveograph indices and GI, these tests should be carried out on samples from different environments [33]. Moreover, some authors [48,68] pointed out that Alveograph indices do not seem to distinguish the contribution of the amount of protein from its quality. In other words, a high value for Alveograph strength (W) may be related either to the high percentage of protein or to the high quality of the protein network [69]. This issue seems to be obviated when gluten viscoelasticity is assessed by the Glutograph test. Correlated with SDS, GI, W, and P values, the stretching value is a good indicator of gluten strength (Table S1). However, analyzing extracted gluten instead of semolina dough might ’hide’ the potential role of other compounds—as well as their interactions with proteins—in defining the technological potential of semolina samples.

Among the GlutoPeak indices, the area under the curve—which takes into consideration both the peak torque and peak time—seems to be the most indicative index, since it is correlated with W index. As regards the GSI proposed by Sissons and Smit [62], although correlated with dough strength (W), correlations with pasta cooking quality remain to be investigated. In this context, some studies showed a negative correlation between PMT and pasta stickiness and bulkiness [42,70]; although, more samples need to be evaluated.

Although all operators in the durum wheat supply chain support the use of rheological tests for predicting the quality of cooked pasta, only a few studies showed relationships between semolina and pasta quality. As regards the GI, it is positively correlated with cooked firmness [33]. According to Alamri et al. [71] some Glutograph indices are positively correlated with cooking quality (i.e., stretching time versus cooking loss and firmness), whereas others exhibit a negative correlation (i.e., relaxation versus cooking loss). Alveograph indices are the most frequently related to pasta cooking behavior. In particular, the W parameter was significantly correlated with firmness tested by devices for texture analysis [33] as well as the quality judgment expressed by a trained panel [7,32]. Among the latest approaches, GlutoPeak test measurements of maximum torque and energy provided information on firmness, stickiness, and bulkiness of cooked pasta [40,70]. The suitability of this rheological approach in predicting the stickiness of cooked pasta was also confirmed by Sissons [70]. Finally, although the quantity and quality of the proteins in the raw material are universally considered the crucial properties in determining the cooking quality of pasta, the role of starch in cooking behavior should not be forgotten. This is confirmed by the results of D’Egidio et al. [25], which highlight how the C3 parameter (related to starch gelatinization) of Mixolab test is negatively correlated to bulkiness and to the overall judgment of the pasta by a trained panel. The number of analyzed samples (generally low), differences in the characteristics of the raw materials (i.e., gluten content and quality, as well as amylose content) differences in pasta-making conditions (e.g., extrusion pressure, and drying temperature), in cooking procedures (ratio pasta:water, optimal or pre-fixed cooking time, etc.) and in methods used for cooked pasta evaluation (sensory evaluation by trained personnel or devices) among the studies might account for the difficulty in determining relationships between semolina and pasta quality.

## 5. Conclusions

The assessment of semolina quality continues to be of interest to researchers and pasta companies, suggesting that the ideal test to determine pasta-making potential has not yet been found. However, in the last few years, numerous efforts have been made to propose rapid and reliable tests for semolina quality. For most of them, the lack of a standard method limits their diffusion. Furthermore, some tests are based on the evaluation of the gluten extracted from the dough; this approach is controversial as the extraction of a component can alter and modify real interactions between the different (macro)molecules of the “native” system. Moreover, almost all the rheological tests adopted so far in the pasta-making sector derive from tests developed for bread-making using common wheat flour, simulating the phenomena occurring in that process. In addition to the different particle size between semolina and flour, which is not a secondary parameter in influencing the rheological behavior of a raw material, the conditions adopted in pasta-making and bread-making are very different, both in terms of hydration level during kneading, and of the type and intensity of physical stresses developing during processing. Thus, the relation between the rheological properties of semolina and pasta quality is often weak. Furthermore, although the positive correlations between rheological properties and cooking quality reported by some authors are significant, they remain relatively weak, indicating the considerable variation between measurements to test quality and pasta quality as perceived by consumers. It should be noted that the studies that have been carried out on durum wheat varieties and pasta are generally lab-scale in dimension, whereas industrially, a mixture of varieties is processed into making pasta.

In addition, since each test addresses a specific gluten property (e.g., tenacity, elasticity, etc.), several authors have proposed a correlation among the indices obtained by the various approaches. A multivariate approach might help in identifying which attributes best differentiate pasta samples according to gluten quality. In addition to gluten, starch is also involved in determining pasta quality. Thus, the relation between pasta quality and semolina pasting properties should be taken into consideration in further studies.

Over the years, breeding programs have improved the qualitative characteristics of durum wheat, resulting in varieties that are increasingly rich in proteins, making for very strong dough [72]. Consequently, the rheological tests reported in the literature for semolina may have used raw materials of poor quality. Therefore, a fast, reliable approach to predict the behavior of durum wheat semolina and the cooking quality of its corresponding pasta needs to be elaborated.

## Figures and Tables

**Table 1:** The main attributes used to describe the properties of gluten of durum wheat dough.

Gluten Property	General Definition	Applied to Durum Wheat Dough and Pasta
Viscoelasticity	Ability of solids to have simultaneous viscous and elastic properties	The determinantal characteristic of gluten, necessary for pasta-making process
Viscosity	Resistance of a liquid to flow	It determines in which way the dough flows through the press and the dye
Elasticity	Ability of solids to recover their initial shape after deformation	It allows the mass to withstand strong compression (about 10 MPa) during the extrusion phase and to assure regular shrinkage during drying (shape maintenance)
Extensibility	Maximum degree of deformation reached by solids before breakage	Excessive extensibility doesn’t counteract the mechanical stresses during processing
Tenacity	Resistance of dough to deformation	It allows the mass to resist, without breaking, the high/intense mechanical stresses (shear and stretching) occurring during the extrusion phase
Strength	Ability of solids to resist mechanical stress	It allows proteins to form a regular and continuous network that promotes good cooking quality

**Table 3:** Main indices provided by the rheological approaches used for semolina characterization.

Test	Index	Description	Type of Information
Gluten Index	Value from 0 to 100	Percentage of wet gluten retained in the sieve	Gluten strength
Glutograph®	Stretching time	Time to reach deflection or value after time threshold (shear/stretch angle)	Gluten extensibility
	Relaxation	Recovery angle after 10 s of stress removal	Gluten elasticity
Alveograph	P	Maximum pressure (mmH_2_O) needed to deform the dough till breakage	Dough tenacity
	L	Length of the curve (mm)	Dough extensibility
	P/L	Ratio between P and L	Balance between dough tenacity and extensibility
	W	Energy (in 10^−4^ J) required for dough deformation till breakage; area under the curve	Dough strength
	Ie	Ratio between P200 (i.e., the pressure 4 cm from the beginning of the curve) and the value of P	Dough elasticity
GlutoPeak®	Maximum consistency (BEM)	Maximum height of the peak	Consistency of gluten upon aggregation
	Peak maximum time (PMT)	Time required to reach the maximum height	Time for gluten aggregation
	Aggregation energy	Area from 15 s before to 15 s after the maximum peak	Gluten strength
	Total energy	Area from 0 s before to 15 s after the maximum peak	Gluten strength
Mixolab	Water absorption	Amount of water to add to semolina to reach an optimal consistency of 1.10 Nm (C1)	The higher the value, the higher protein quantity/quality
	Development time	Time needed to reach C1	The higher the value, the higher protein quantity/quality
	Stability	Time around C1 where the torque is higher or equal to the real value of C1–C1*11%	Dough resistance to mixing
	Torque C2	The lowest point of the curve when the device starts heating the dough	Weakening of protein
	C1–C2	Difference between Torque C1 and C2	Gluten strength
	Torque C3	The maximum torque obtained after C2 during the heating phase.	Starch gelatinization
	Torque C4	The minimum torque after the holding period at 90°C	Stability during heating and mixing
	Torque C5	Torque at the end of the test	Starch retrogradation tendency

P, maximum pressure; L, maximum length; P/L, pressure:length ratio; W, area under the curve; Ie, P200/P (P200: pressure at 4 cm from the beginning of the curve).

## Supplementary Materials

The following are available online at https://www.mdpi.com/article/10.3390/foods10122947/s1. Table S1: Pearson’s correlation coefficients between the rheological indices used to define semolina quality.
